# Short-chain oat fiber improves gastrointestinal tolerance and regulates glucose metabolism: a two-week open-label study in healthy adults

**DOI:** 10.3389/fnut.2026.1745303

**Published:** 2026-06-10

**Authors:** Angela M. Marcobal, Katharine M. Ng, Riley A. Drexler, Bruce R. McConnell, Matthew J. Amicucci

**Affiliations:** One Bio Inc., Sacramento, CA, United States

**Keywords:** functional fiber, gastrointestinal tolerance, glucose control, oat fiber, postprandial glucose, β-glucan

## Abstract

**Introduction:**

Fiber intake is the most common nutritional inadequacy in the Western diet, with most adults consuming less than half of the recommended intake and only 5% meeting the RDI. A novel, short-chain β-glucan derived from oats (scOat fiber), with improved solubility, low viscosity and enhanced palatability compared to conventional oat fibers, was investigated for its benefits as a source of fiber supplementation.

**Methods:**

A 14-day pilot study evaluated the gastrointestinal tolerance and functional benefits of scOat fiber in 63 healthy adults assigned to receive 5, 10 or 20 g daily doses. The primary outcome, gastrointestinal tolerability, was assessed using the Gastrointestinal Symptom Rating Scale (GSRS). Secondary outcome included glycemic response during rice challenges, evaluated in a subgroup of 38 participants using continuous glucose monitoring (CGM). CGM was also used to explore overall glucose dynamics. Additional exploratory outcomes (mood, energy, appetite and sleep) were assessed via validated questionnaires.

**Results:**

scOat fiber was exceptionally well tolerated across all doses, with no increase in GSRS scores, which remained in the low to mild range. Significant reductions in total GSRS scores were observed, with benefits evident after 1 week at 5 g/day and maintained over time at both 5 and 10 g/day groups. Evaluation of GSRS sub-categories revealed that the 5 g/day and 10 g/day dose groups experienced significant reductions in abdominal pain symptoms. Both dose groups also demonstrated a significant decrease in constipation at the end of the study. Postprandial glucose responses were attenuated following product use, with a significant reduction in peak glucose during rice challenges after 2 weeks in the 20 g/day group. Both 10 and 20 g/day doses were associated with significant improvement in glycemic metrics, including reductions in glucose mean, all glycemic excursions, and an increase in time-in-range. Exploratory analysis suggested that scOat Fiber may improve mental health and concentration in participants with elevated baseline symptoms.

**Conclusion:**

Despite the lack of a placebo control and short duration, the dose-dependent nature of the results supports the potential of scOat Fiber as a well-tolerated and functional source of fiber with benefits including glycemic control, digestive health and mental health.

**Clinical trial registration:**

https://clinicaltrials.gov/study/NCT06739941, Identifier: NCT06739941.

## 1 Introduction

Dietary fiber plays a key role in contributing to and maintaining human health through various pathways such as modulating glycemic response, cholesterol levels, blood pressure, mineral absorption, gut barrier function and the gut microbiota (1–3). Conversely, a lack of dietary fiber intake is associated with many chronic conditions (4–6). However, according to the 2020–2025 *Dietary Guidelines for Americans*, only 10% of women and 3% of men consume the recommended daily fiber intake, and in general, the adult population in the US consumes less than 50% of adequate fiber amounts (7). This fiber gap is further exacerbated by growing popularity of low carbohydrate diets, such as the ketogenic, paleo or carnivore diets, which often restrict or eliminate fiber-rich plants including whole grains in favor of animal-based proteins and fats (7).

With 340 million people in the United States running a 16 g/day gap in dietary fiber intake, this presents a 2 billion kilogram opportunity for the food, beverage and supplement industries to develop fiber enriched products that can dramatically improve public health through the reduction of chronic disease and all-cause mortality (8). However, challenges in formulation and gastrointestinal (GI) tolerance issues have limited the use of natural dietary fibers in ready-to-eat and drink food products. Most dietary fibers isolated from natural plant sources or food industry side streams have physical characteristics that complicate their formulation, such as gritty texture and tendency to gel, leading to undesirable changes in organoleptic and sensory properties (9). As a result, modified or synthetic fibers, such as corn fiber, wheat dextrin or cassava root fiber, have been increasingly used in foods and supplements (10). Inulin, commonly extracted from chicory root, agave, or Jerusalem artichoke, is a popular alternative that is more compatible with the formulation needs of the food industry. However, when formulated into food and beverages with acidic pH, inulin is chemically fragile, breaking down into fructose and consequently increasing sugar content (11). In addition, the tolerability of inulin is a concern. Five to ten g/day doses of inulin are required to achieve health benefits, but they are frequently associated with GI side effects, including gas, bloating, and abdominal pain (12, 13). Also, recent studies have associated the consumption of high concentrations of inulin with inflammation in sensitive individuals (14, 15).

Mixed linkage β-glucans, are polysaccharides naturally present in several grains such as oat, barley, rye and wheat (16) and present an opportunity for fiber supplementation. As human digestive enzymes are unable to cleave the β1-4 and β1-3 glucose linear bonds of β-glucans, these structures pass into the colon where they are metabolized by the gut microbiota (17). Cereal β-glucans have been acknowledged by regulatory bodies for their positive impact on cardiovascular and metabolic health (18). However, oat β-glucans form highly viscous gels, and their thick mouthfeel makes them unsuitable for many applications, especially beverages. β-glucans are also stable over most conditions used in food and beverages (19).

To exploit the benefits of oat fiber while mitigating its formulation issues, a novel, short-chain oat fiber product (scOat Fiber), obtained by depolymerization of natural oat β-glucan, has been developed. Traditionally, the physiological benefits of long-chain oat β-glucans improving blood glucose profiles are attributed to their high viscosity, which delays gastric emptying and slows nutrient absorption in the small intestine. In contrast, the depolymerized short-chain form exhibits minimal viscosity yet retains distinctive functional properties. It has recently been shown to exert a prebiotic effect on human microbial communities, with robust production of short chain fatty acids (SCFA) (20). *In vitro* fecal fermentations using multiple human stools, revealed an outstanding fermentability profile and high production of beneficial SCFAs, including butyrate. Additionally, the impact of this novel fiber product on mechanisms related to glucose control, such as inhibition of key digestive enzymes and sodium glucose co-transporters (SGLT-1), have been demonstrated *in vitro*, suggesting a capacity to slow down carbohydrate digestion and glucose uptake (21).

Although the GI tolerance of scOat Fiber is expected to be similar to native oat fiber, until now, this has not been shown. Further, the potential benefits suggested by preclinical studies have not been validated in humans. The aim of this dose ranging study in healthy adults was to assess the tolerability, potential digestive health and metabolic effects of the novel oat fiber ingredient in a real-world setting. Participants were asked to consume three different doses (5, 10 and 20 g/day), for 2 weeks. The doses were chosen to reflect a range of realistic applications in products, and to assess tolerability at a high daily dose. The high daily dose was at a level that normally exceeds the GI discomfort threshold seen with other fibers, such as inulin or psyllium husk. This study also explored outcomes including mood, energy, appetite, and sleep. The study was also intended to inform the design of a future, large, controlled study.

## 2 Materials and methods

### 2.1 Study design and conduct

This study was a prospective, open-label, three-arm clinical study to evaluate the GI tolerance and safety of daily oral intake of 5 g, 10 g and 20 g doses of scOat Fiber in healthy adults. The scOat Fiber (commercially available as one.bio Oat Fiber) was manufactured by One Bio Inc. (Sacramento, CA, United States) by depolymerizing oat fiber. The scOat Fiber ingredient comprises 91.3% oat fiber, and minor residual starch and free sugar (<3%). The oat fiber fraction contains 90.4% β-glucan. Most oligosaccharides have short-chain lengths of 3 to 30 monomer units. The study was conducted by People Science Inc., in accordance with the ethical principles outlined in the Belmont Report: Ethical Principles and Guidelines for the Protection of Human Subjects of Research (United States National Commission for the Protection of Human Subjects of Biomedical and Behavioral Research, April 18, 1979) and the Declaration of Helsinki. The study was conducted according to the United States Code of Federal Regulations Title 45 and State of California Health and Safety Code. The protocol was approved by the Advarra IRB (Columbia, MD, United States; Protocol ID#Pro00074413) and registered with ClinicalTrials.gov (NCT06739941). All participants were recruited from the community through social media channels and researcher networks and all provided written informed consent prior to starting the study.

After inclusion in the study, the participants completed an informed consent form and provided demographic information, medical history, dietary habits, and physical measurements. Complete Blood Count (CBC), Comprehensive Metabolic Panel (CMP), and hemoglobin A1C tests were performed at a Quest Lab to assess study eligibility. Eligible participants were then sequentially assigned upon enrollment to one of three product dose groups (5 g, 10 g, or 20 g of scOat Fiber per day), consistent with the exploratory open-label design of the study. The scOat Fiber was provided as a free-flowing powder to be dissolved in a glass of water immediately before consumption. Each daily dose was packaged in individual sachets (5, 10 or 20 g per sachet) and consumed once per day. The product was shipped directly to participants under ambient conditions. The study concluded with follow-up blood tests.

The validated Gastrointestinal Symptom Rating Scale (GSRS) was used to evaluate tolerability of scOat Fiber (22). GSRS scores are grouped into five sub-categories (reflux, abdominal pain, indigestion, diarrhea, and constipation) or summarized as a total score. The GSRS questionnaire was completed at intervention T0, after 1 week of fiber consumption (intervention T2), and at the end of the study (intervention T3). Participants also completed:

1. A weekly questionnaire with 12 questions assessing appetite, anxiety, depression and concentration. This weekly questionnaire consisted of a survey drawn from custom questions, and a subset of items in the validated Generalized Anxiety Disorder 7-Item (GAD-7) and the Patient Health Questionnaire 9-Item (PHQ-9) (23, 24).
2. A daily questionnaire consisting of five questions that enquired about (i) presence or absence of GI symptoms, (ii) whether an unusual meal was consumed (to potentially explain unusual symptoms, glucose peaks), (iii) unusual drops in energy, (iv) sleep quality, and (v) time taken to fall asleep.
3. A daily adverse event questionnaire to report on any negative effects they may have experienced during product consumption.

All participants also completed a standardized at-home rice challenge to evaluate within subject changes in postprandial glucose responses over time. The rice challenge served as a standardized reference meal repeated at predefined timepoints, allowing each participant to serve as their own control, and did not involve randomization. The challenge involved consuming 210 g (7.4 oz) of ready-to-eat cooked rice (NISHIKI® brand), providing approximately 75 g of available carbohydrate, after an overnight fast of 8–10 h. The rice was consumed alone prior to commencing scOat Fiber use (intervention T0), and in combination with scOat Fiber at the start of scOat Fiber consumption (intervention 1, T1), after 1 week of daily scOat Fiber consumption (intervention 2, T2), and after 2 weeks of daily scOat Fiber (intervention 3, T3). Participants wore a Dexcom G6 Pro Continuous Glucose Monitoring (CGM) device for 19 days (baseline period and intervention period) to evaluate blood glucose dynamics using multiple metrics. These devices were used continuously and collected data approximately every 5 min.

**Figure 1:**
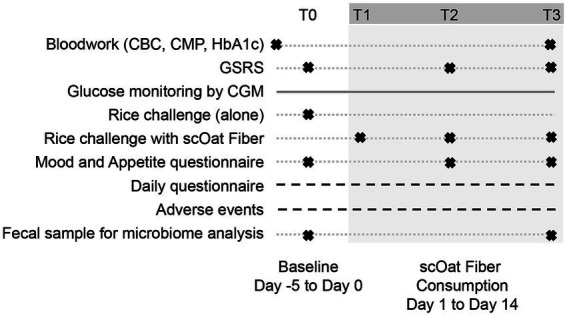
Clinical study design. Data was collected continuously (gray line), daily (dashed line), and at specified timepoints (dotted lines). Intervention times are shown as T0 (rice challenge alone), T1 (at the start of fiber consumption), T2 (after 1 week of daily fiber consumption), and T3 (after 2 weeks of daily fiber consumption). Daily questionnaire includes information related to absence of GI symptoms, unusual meals, unusual drop of energy, sleep quality, and time to fall asleep.

An overview of the study design is shown in Figure 1. All data were collected and securely stored on a platform (Chloe, Consumer Health Learning & Organizing Ecosystem) run by People Science Inc. on Amazon Web Services HIPAA-compliant servers.

### 2.2 Inclusion and exclusion criteria

Participants were eligible for participation in the study if they were aged between 18 years and 70 years, were able to read and understand English, understand and provide informed consent, use a personal smartphone device and to complete study assessments over the course of up to 7 weeks. Exclusion criteria were defined as follows. Participants without a smartphone and/or internet access, had any significant abnormal laboratory value seen on CBC and CMP testing and/or an A1C ≥ 6.5%, were diagnosed with a GI, digestive or metabolic disease or disorder including Crohn’s disease, ulcerative colitis, irritable bowel syndrome, celiac disease, gluten allergy, diabetes, or obesity, or an illness, disease or condition which, in the opinion of the principal investigator, may have impacted their ability to participate in the study or impacted the study outcomes, had any known allergic reaction to any component of the scOat Fiber or the rice challenge product, were prescribed medication likely to influence the study measures, had consumed fiber supplements 30 days prior to enrollment or commenced consumption at any time during the study, had undergone a major change in diet or exercise 30 days prior to enrollment or who initiated such a change at any time during the study, had consumed antibiotics 30 days prior to enrollment and who initiated such consumption at any time during the study, were pregnant, wished to become pregnant, or who were breastfeeding, had excessive alcohol use or substance abuse, were unlikely for any reason to be able to comply with the study protocol or who were considered unsuited for participation in the study by the principal investigator.

### 2.3 Study outcomes

The primary outcome was GI tolerability of the three different doses of scOat Fiber as measured by change in mean total score on the GSRS at interventions T2 and T3 with respect to Intervention T0.

Secondary and exploratory outcomes included:

1. The effects on GI symptoms of the three different doses of scOat Fiber as measured by change in mean total score, and the 5 sub-category scores on the GSRS at interventions T2 and T3 with respect to Intervention T0.
2. The effects of a single intervention of the three different doses of scOat Fiber on postprandial glucose uptake after a white rice challenge, as measured by magnitude-based metrics peak glucose, glucose spike height, total area under the curve (AUC), and incremental AUC (iAUC) using a CGM device at intervention T0 and intervention T1.
3. The effects of 2 weeks of intervention of the three different doses of scOat Fiber on postprandial glucose uptake after a white rice challenge, as measured by magnitude-based metrics peak glucose, glucose spike height, AUC and iAUC using a CGM device at intervention T0 and intervention T3.
4. The effects of the three different doses of scOat Fiber on glucose uptake dynamics as measured by a CGM device used during baseline and throughout product use period.
5. The effects of the three different doses of scOat Fiber on appetite, anxiety, mood, irritability, anhedonia, concentration and sleep as measured by survey questions.

Safety was assessed based on CBC and CMP test results and monitoring adverse events (AEs) throughout the course of the study.

### 2.4 Statistical analysis

All data analyses were conducted using GraphPad Prism (GraphPad Software, Boston, MA). Normality was assessed using the Shapiro–Wilk (SW) test. Parametric statistics (ANOVA, Student’s *t*-tests) were used to evaluate differences between normally distributed measures. Non-parametric statistics were used in cases where data were not normally distributed (Friedman’s Test for within-doses across time points, Wilcoxon for post-hoc analyses). The Two-stage step-up method of Benjamini, Krieger and Yekultieli was used to control the False Discovery Rate in the case of multiple comparison testing. Mann-Kendall (MK) tests were used to evaluate potential trends over time in daily data. Fisher’s Exact Test (FET) was used to evaluate potential differences in % improvers.

For the primary outcome, data from all participants who completed intervention T1 were used in the analysis, regardless of whether they completed the study. The same approach was taken for the secondary outcome relating to effects on GI symptoms. For the blood glucose related outcomes, participants were considered eligible for analysis if they completed the rice challenge tests at interventions T0 and T3 and had at least 90% CGM data coverage during each rice challenge. Data missing at interventions T1 and T2 were imputed using linear projection. The glucose baseline was defined as the average blood glucose value during the 2 h fasting period prior to rice consumption. Glucose magnitude-based metrics, including peak glucose, glucose spike height, AUC, and iAUC were calculated using Simpson’s Rule (i.e., trapezoids with parabolic upper edges (18–20)). These metrics were aggregated across participants for each rice challenge test day. The peak glucose was defined as the highest value recorded within 4 h after rice intake. The glucose spike height was calculated as the difference between the peak glucose and glucose baseline. To evaluate overall impact on glycemic control, the blood glucose data were analyzed longitudinally. The analysis included only participants with complete CGM data across all assessed time points. Metrics were calculated for the baseline period (prior to intervention T1) and for the final 5 days of product use before Intervention 2 and before intervention 3. Metrics for glycemic control included glucose mean, standard deviation (SD), % coefficient of variation (% CV), excursion size of all excursions, and mean amplitude of glycemic excursions (MAGE). MAGE was calculated per individual on a daily basis as previously described (25, 26), then averaged across days and participants. In addition, time in range (TIR) was assessed using both conventional (TIR, 70–140 mg/dL) and a more stringent ideal time in range (iTIR 72–110 mg/dL).

## 3 Results

### 3.1 Sample size and demographics

67 participants were enrolled and sequentially assigned to one of three dose groups (5 g, 10 g and 20 g/day), and 63 were evaluated (5 g/day, *n* = 24; 10 g/day, *n* = 19; 20 g/day, *n* = 20). Three participants were withdrawn due to individual participant decisions unrelated to the scOat Fiber, and one participant was withdrawn due to an adverse event related to GI symptoms. Demographics are described in Table 1.

**Table 1:** Study demographics.

Category	Total % (count)
Sex	
Female	57.1 (36)
Male	42.9 (27)
Ethnicity	
Caucasian	63.5 (40)
Asian American	19.0 (12)
African American	9.5 (6)
Hispanic	3.2 (2)
Other	4.8 (3)

### 3.2 Safety and tolerance of scOat fiber

scOat fiber was well tolerated across all groups, with no increase for any of the three doses groups in total GSRS scores, in any of the five GSRS domain scores, and in any of the 15 GSRS symptom scales, over the course of the study. A difference between the groups was detected (Kruskal-Wallis, *p* = 0.0268), driven by a combination of symptom improvement in two of the groups, and differences in baseline mean scores.

Reported adverse events were predominantly mild and included gas (*n* = 26 participants), bloating (*n* = 15 participants), constipation (*n* = 13 participants), abdominal pain (*n* = 7 participants) and diarrhea (*n* = 5 participants) (Supplementary Table S2). The number of AEs did not exhibit any dose-dependent relationship. These symptoms were transient, not dose limiting, and none were assessed to be clinically significant. All adverse events were assessed to be possibly related to scOat Fiber or the rice challenge product. One participant discontinued the study due to GI symptoms after intervention 1 (i.e., after first consumption of scOat Fiber). The participant was found to be concurrently consuming the antiemetic medication Zofran (Ondansetron). In view of the concurrent use and known side effects of Zofran, no conclusive link between the scOat Fiber and the adverse effect could be established, and the relationship was rated as “Possible.” The participant fully recovered.

Analysis of the blood samples revealed no irregularities considered due to the intake of the scOat Fiber in any dose group. All parameters measured remained within the normal range throughout the intervention and any differences that were statistically significant, were not considered to be clinically relevant (data not shown).

Taken together, these data demonstrate that scOat Fiber is safe and well tolerated at daily intakes up to 20 g/day.

### 3.3 Effects on gastrointestinal symptoms

The participants were healthy adults with no or very low GI symptoms when entering the study. Despite this, the 5 and 10 g/day dose groups exhibited significant improvement in GI symptoms over time, shown by a decrease in total GSRS scores (Table 2, Friedman *p* = 0.0012 and *p* = 0.0105, respectively). *Post hoc* testing revealed that the 5 g/day group had a significant improvement in Total GSRS score between intervention T0 and interventions T2 and T3 (Table 2, FDR-Adjusted Wilcoxon, *p* = 0.0009, *p* = 0.0026, respectively). Similarly, the 10 g/day group showed improvement in total GSRS between intervention T0 and T3 (Table 2, FDR-Adjusted Wilcoxon, *p* = 0.0124). The total GSRS score for the 10 g/day group at intervention T0 was significantly lower than that of the 5 g/day group (Kruskal-Wallis, *p* = 0.0187), which may have limited the ability to detect symptom improvement between T0 and T2.

**Table 2:** GSRS total scores across the study period.

Group	*n*	Intervention T0	Intervention T2	Intervention T3	Friedman’s *p*	Wilcoxon’s *p* (T0 to T2)	Wilcoxon’s *p* (T0 to T3)
5 g/day	24	1.82 (0.58)	1.48 (0.41)	1.44 (0.52)	**0.0012**	**0.0009**	**0.0026**
10 g/day	19	1.53 (0.50)	1.44 (0.51)	1.26 (0.27)	**0.0105**	0.4533	**0.0124**
20 g/day	20	1.65 (0.72)	1.59 (0.66)	1.71 (0.95)	0.6328	0.4654	>0.9999

Values represent mean (SD) for each group receiving scOat fiber at different doses. Statistical significance of changes over time (Friedman’s *p*-value) and between intervention T0 and interventions T2 and T3 (FDR-adjusted Wilcoxon’s *p*-value) are reported as *p*-values. Significant results (*p* < 0.05) are highlighted in bold.

When analyzing the GSRS domains, the 5 g/day and 10 g/day dose groups exhibited a significant reduction of abdominal pain symptoms over time (Friedman, *p* = 0.0005 and *p* = 0.0074, respectively, Supplementary Table S1). *Post hoc* testing revealed that the 5 g/day group exhibited a significant reduction of abdominal pain symptoms between intervention T0 and interventions T2 and T3 (FDR-adjusted Wilcoxon, *p* = 0.0012 and *p* = 0.0122, respectively). The 10 g/day group experienced a significant reduction of abdominal pain at intervention T3 compared with intervention T0 (FDR-adjusted Wilcoxon, *p* = 0.0187). Additionally, a significant decrease in constipation symptoms was observed over time for the 5 g/day and 10 g/day dose groups (Friedman, *p* = 0.0408 and *p* = 0.0204), and a pairwise significance was found in post-hoc comparisons of intervention T0 and intervention T3 for the 10 g/day groups (Wilcoxon, *p* = 0.0244). No change over time in the other four GSRS domain scores was detected, but a significant decrease of indigestion symptoms was found for the 5 g/day dose group when comparing intervention T0 vs. intervention T2 (FDR-adjusted Wilcoxon, *p* = 0.0465, Supplementary Table S1).

Given that bloating and passing gas are common complaints for fibers, these GSRS scales were analyzed separately from the indigestion domain they are part of. No increase in symptoms was detected in any of the groups at any intervention time point. The 5 g/day group in fact exhibited an improvement over time in passing gas symptoms (Friedman, *p* = 0.026).

### 3.4 Impact of scOat fiber on postprandial glucose

A total of 38 participants were included in the analysis across the three dose groups: 5 g/day (*n* = 13), 10 g/day (*n* = 13), and 20 g/day (*n* = 12). Glucose data from 25 study participants were excluded from the analysis due to (a) missing baseline glucose challenge data (*n* = 6), (b) self-reported failure to fast (*n* = 2), (c) improper reporting of rice challenge timing (*n* = 2), and (d) absence of glucose data (*n* = 15). Blood glucose metrics did not differ between the dose groups at intervention T0 (*p* > 0.05), data not shown.

**Figure 2:**
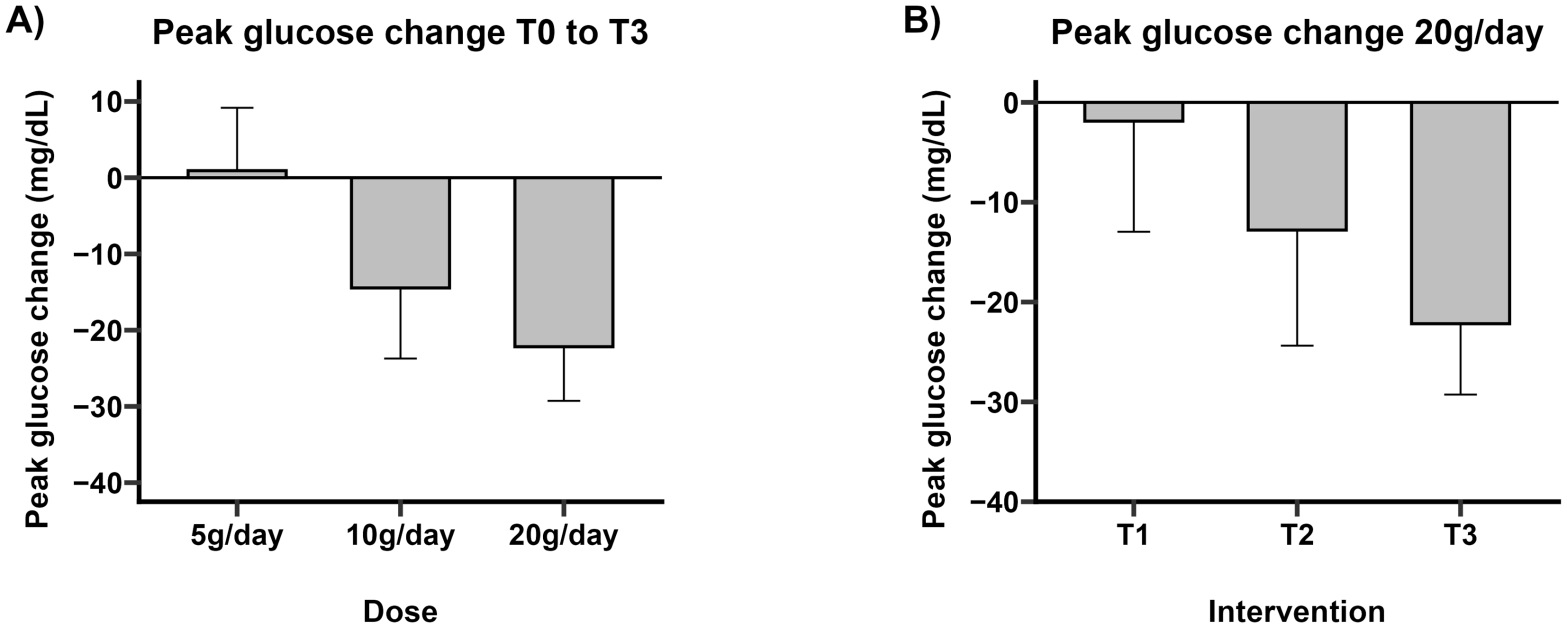
Effect of scOat fiber on peak glucose during rice cereal challenges. **(A)** Change of peak glucose during rice challenges performed at intervention T3 vs. intervention T0 in all participants; **(B)** Change in peak glucose during rice challenges performed at interventions T1, T2, and T3 in 20 g/day group. Error bars represent standard error of the mean.

To understand the dynamics of postprandial blood glucose response, peak glucose and spike height were measured after rice consumption. A general downward trend in peak glucose over time was observed, suggesting that continued scOat Fiber intake may moderate glycemic responses. The effect was most evident at higher intake levels, with notable reductions at 20 g/day (Figure 2A). Moreover, this attenuation of glycemic excursions appeared to intensify with both dose and intervention duration, as reflected by the progressive decreases across T1, T2, and T3 in the 20 g/day group (Figure 2B). A significant reduction in peak glucose between interventions T0 and T3 was revealed in the 10 and 20 g/day groups (FDR-adjusted Student’s *t*-test, *p* = 0.0227 and 0.0177; Table 3). Furthermore, when comparing peak glucose during rice challenge at intervention T3 vs. rice challenge at intervention T0, a reduction was observed in 54% of participants from the 5 g/day group, 69% of participants from the 10 g/day group and 83% of participants from the 20 g/day group.

**Table 3:** Peak glucose and glucose spike height during rice challenges.

Group	Intervention	Peak glucose (mg/dL)	Spike height (mg/dL)
5 g/day, *n* = 13	T0	167.15 (32.66)	59.58 (32.14)
	T1	159.85 (35.42)	51.38 (33.23)
	T2	171.08 (34.96)	59.69 (39.75)
	T3	168.08 (34.32)	57.96 (34.29)
	ANOVA *p*	0.5862	0.7206
	Student’s *p* T0 vs. T1	0.2525	0.2932
	Student’s *p* T0 vs. T2	0.6443	0.9901
	Student’s *p* T0 vs. T3	0.9162	0.8470
10 g/day, *n* = 13	T0	174.15 (35.14)	67.04 (28.55)
	T1	164.62 (36.86)	65.62 (30.88)
	T2	173.46 (28.50)	74.15 (23.54)
	T3	159.69 (24.09)	63.58 (23.67)
	ANOVA *p*	0.0887	0.4738
	Student’s *p* T0 vs. T1	0.0947	0.8379
	Student’s *p* T0 vs. T2	0.5434	0.2943
	Student’s *p* T0 vs. T3	**0.0227**	0.6180
20 g/day, *n* = 12	T0	166.33 (25.60)	61.50 (26.50)
	T1	164.46 (34.10)	59.77 (28.78)
	T2	153.54 (25.90)	51.92 (31.01)
	T3	144.17 (15.47)	41.92 (16.29)
	ANOVA *p*	0.0979	0.2856
	Student’s *p* T0 vs. T1	0.6353	0.8861
	Student’s *p* T0 vs. T2	0.3428	0.4737
	Student’s *p* T0 vs. T3	**0.0177**	**0.0241**

Values represent mean (SD) for each group receiving scOat Fiber at different doses. Statistical significance of changes at intervention period compared to Intervention T0 (ANOVA, FDR-adjusted Student’s *p*-value) are reported. Significant results (*p* < 0.05) are highlighted in bold.

Similar outcomes were seen for the glucose spike height. The glucose spike height showed a similar dose-dependent reduction, with the 20 g/day group demonstrating a significant decrease in glucose spike height between interventions T0 and T3 (FDR-adjusted Student’s *t*-test, *p* = 0.024; Table 3).

Additionally, postprandial glycemic exposure was assessed using total AUC and iAUC during rice challenges, at intervention T1, intervention T2 and intervention T3, compared to the rice challenge at intervention T0. Total AUC and iAUC of each group generally trended toward lower values at interventions T2 and T3, although statistical significance was not found for any dose or at any intervention (ANOVA, Student’s *t*-test; Table 4).

**Table 4:** Incremental (iAUC) and total area under the curve (AUC) glucose responses during rice challenges.

Group	Intervention	iAUC	Total AUC
5 g/day, *n* = 13	T0	84.63 (61.45)	498.92 (86.62)
	T1	94.55 (57.28)	467.82 (80.81)
	T2	92.58 (75.69)	459.24 (124.75)
	T3	66.65 (56.37)	495.32 (86.64)
	ANOVA *p*	0.3571	0.8676
	Student’s *p* T0 vs. T1	0.2546	0.3182
	Student’s *p* T0 vs. T2	0.7041	0.29996
	Student’s *p* T0 vs. T3	0.5434	0.8563
10 g/day, *n* = 13	T0	72.64 (50.49)	474.51 (71.65)
	T1	82.06 (53.30)	475.96 (100.70)
	T2	58.78 (35.30)	471.76 (63.34)
	T3	44.09 (33.97)	459.51 (50.94)
	ANOVA *p*	0.1719	0.9051
	Student’s *p* T0 vs. T1	0.6741	0.9704
	Student’s *p* T0 vs. T2	0.3184	0.9242
	Student’s *p* T0 vs. T3	0.1685	0.4492
20 g/day, *n* = 12	T0	83.42 (48.05)	478.16 (69.97)
	T1	92.10 (45.89)	480.91 (57.32)
	T2	97.33 (56.64)	471.13 (60.48)
	T3	81.35 (46.66)	434.85 (34.80)
	ANOVA *p*	0.7291	0.2469
	Student’s *p* T0 vs. T1	0.8744	0.9256
	Student’s *p* T0 vs. T2	0.5271	0.8121
	Student’s *p* T0 vs. T3	0.6353	0.0601

Values represent mean (SD) for each group receiving scOat fiber at different doses. Statistical significance of changes at intervention period compared to Intervention T0 (ANOVA, FDR-adjusted Student’s *p*-value) are reported.

Together, these findings suggest that scOat Fiber may exert a dose and time dependent effect on postprandial glucose regulation, with the strongest effects observed after 2 weeks at the 20 g/day dose.

### 3.5 Impact of scOat fiber on glucose variability

To evaluate the impact of daily scOat Fiber consumption on glucose variability, metrics including glucose mean, SD, CV, MAGE, TIR, iTIR and all excursions were calculated for the period prior to intervention T1, and 5 days before intervention T2 and 5 days before intervention T3. The analysis included only participants who had complete CGM data across all assessed time points (*n* = 35).

**Figure 3:**
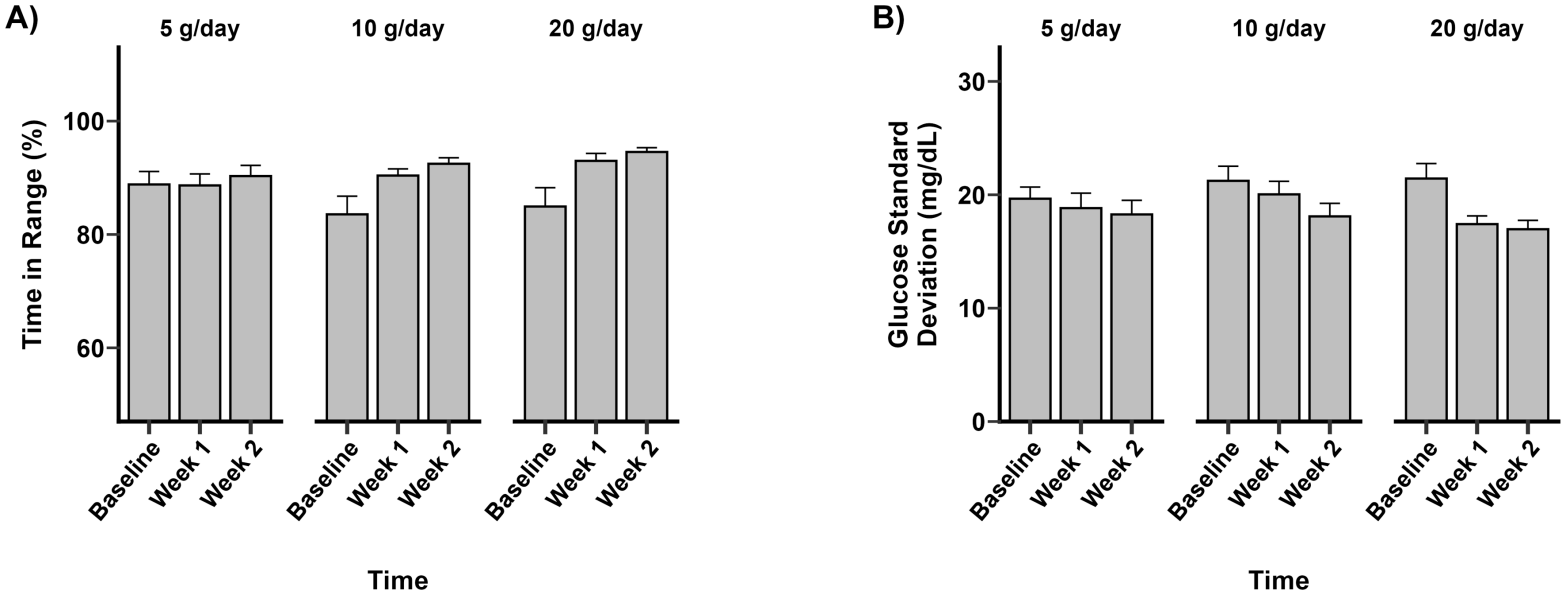
Effect of scOat fiber on glycemic control metrics calculated for the period prior to intervention T1 (baseline) and for the final 5 days of product use before intervention T2 (week 1) and before intervention T3 (week 2) in all participants. **(A)** Change in TIR; **(B)** Change in glucose SD. Error bars represent standard error of the mean.

A significant increase in TIR over time was observed in both the 10 g/day and 20 g/day dose groups (ANOVA, *p* = 0.0377, *p* = 0.0231). TIR increased significantly between interventions T0 and T3 in both 10 g/day and 20 g/day group (FDR-adjusted Student’s *p*-value, *p* = 0.0248 and *p* = 0.0189, respectively). TIR also increased significantly between interventions T0 and T2 in the 20 g/day group (FDR-adjusted Student’s *p*-value, *p* = 0.0393) (Table 5; Figure 3A). Additionally, iTIR increased significantly between interventions T0 and T3 in the 20 g/day dose group (FDR-adjusted Student’s *p*-value, *p* = 0.0411) (Table 5). During the last 5 days before the end of the study and compared with baseline period (before intervention T1), an iTIR increase was observed in 69% of participants from 5 g/day group, 83% of participants from 10 g/day group and 90% of participants at 20 g/day group.

**Table 5:** Glycemic control metrics.

Glycemic control metric	5 g/day	10 g/day	20 g/day
Glucose mean (mg/dL)			
Baseline	112.41 (10.11)	110.22 (12.29)	116.68 (11.97)
Week 1	113.59 (11.30)	103.86 (9.74)	108.96 (6.48)
Week 2	113.50 (10.95)	102.45 (10.75)	108.50 (4.06)
ANOVA *p*	0.1874	0.0684	0.0674
Student’s *p* baseline vs. week 1	0.6274	0.0801	0.0774
Student’s *p* baseline vs. week 2	0.6883	0.6661	0.0619
Glucose standard deviation (mg/dL)			
Baseline	19.65 (3.71)	21.23 (4.47)	21.43 (4.19)
Week 1	18.82 (4.76)	20.04 (3.99)	17.41 (2.30)
Week 2	18.27 (4.48)	18.09 (3.98)	16.96 (2.47)
ANOVA *p*	0.3792	**0.0062**	**0.0005**
Student’s *p* baseline vs. week 1	0.4851	0.8383	0.0736
Student’s *p* baseline vs. week 2	0.2779	**0.0080**	**0.0003**
% coefficient of variation			
Baseline	17.48 (2.88)	19.27 (3.59)	18.36 (3.14)
Week 1	16.68 (4.32)	19.22 (3.16)	15.98 (1.91)
Week 2	16.20 (4.12)	17.58 (3.19)	15.65 (2.32)
ANOVA *p*	0.3332	0.2408	0.0610
Student’s *p* baseline vs. week 1	0.4482	0.9723	0.0791
Student’s *p* baseline vs. week 2	0.2226	0.1730	0.0571
MAGE (mg/dL)			
Baseline	32.83 (7.65)	34.29 (8.94)	34.80 (9.33)
Week 1	33.27 (9.22)	33.92 (7.34)	31.49 (4.40)
Week 2	33.02 (9.66)	30.79 (8.65)	29.59 (5.52)
ANOVA *p*	0.9394	**0.0446**	0.0787
Student’s *p* baseline vs. week 1	0.8548	0.7906	0.1887
Student’s *p* baseline vs. week 2	0.9431	0.0536	0.0526
All excursions (mg/dL)			
Baseline	14.56 (2.63)	16.24 (3.77)	15.89 (2.79)
Week 1	14.07 (3.29)	15.23 (3.43)	13.05 (1.98)
Week 2	13.56 (3.15)	13.89 (3.31)	12.67 (2.01)
ANOVA *p*	0.3825	0.1325	**0.0045**
Student’s *p* baseline vs. week 1	0.5697	0.4570	**0.0133**
Student’s *p* baseline vs. week 2	0.2727	0.0698	**0.0059**
Time in range (iTR, %)			
Baseline	88.82 (8.25)	83.55 (11.08)	84.93 (10.50)
Week 1	88.65 (7.32)	90.39 (4.09)	92.98 (4.17)
Week 2	90.30 (6.80)	92.47 (3.68)	94.55 (2.41)
ANOVA *p*	0.4565	**0.0377**	**0.0231**
Student’s *p* baseline vs. week 1	0.9347	0.0755	**0.0393**
Student’s *p* baseline vs. week 2	0.4126	**0.0248**	**0.0189**
Ideal time in range (iTR, %)			
Baseline	52.38 (19.49)	51.48 (19.15)	47.38 (23.02)
Week 1	49.24 (26.56)	63.38 (22.28)	60.80 (15.22)
Week 2	49.31 (26.88)	66.29 (24.05)	63.71 (9.39)
ANOVA *p*	0.6513	0.0630	0.0734
Student’s *p* baseline vs. week 1	0.5783	0.084	0.1306
Student’s *p* baseline vs. week 2	0.5783	0.0530	**0.0411**

Glycemic control metrics calculated for the period prior to intervention T1 (baseline) and for the final 5 days of product use before intervention T2 (week 1) and before intervention T3 (week 2). Values represent mean (SD), *n* = 38. Bold values indicate significant time-trends (ANOVA, FDR-adjusted Student’s *p*-value, *p* < 0.05).

Also, glucose SD decreased in both 10 g/day and 20 g/day groups (ANOVA, *p* = 0.0062 and *p* = 0.0005, indicating a reduction in overall day-to-day fluctuations. Both 10 g/day and 20 g/day groups also demonstrated a significant decline in SD when comparing interventions T0 and T3 (FDR-adjusted Student’s *p*-value, *p* = 0.0080, *p* = 0.0003) (Table 5; Figure 3B). MAGE values were reduced in the 10 g/day group (ANOVA, *p* = 0.0446), reflecting attenuation of glucose excursions. These findings were further supported by a reduction in the total number of excursions outside of the target range in 20 g/day group (ANOVA, *p* = 0.0045). Significance in the reduction was found when comparing interventions T0 vs. T2, and intervention T0 vs. T3 (FDR-adjusted Student’s *p*-value, *p* = 0.0133 and *p* = 0.0059) (Table 5). Non-significant trends toward reduction in glucose mean and CV was observed for participants receiving 10 g/day and 20 g/day doses.

The results indicate that, in addition to postprandial glucose control, scOat Fiber may have contributed to improved basal glycemia and overall improved glucose control in the 10 g/day and 20 g/day groups.

### 3.6 Exploratory outcomes: mental health, appetite and sleep

To explore the mental health impact of scOat Fiber supplementation, participants completed weekly questionnaires incorporating selected questions from the validated GAD-7 and PHQ-9 assessment tools for mental health.

Due to the high proportion of participants reporting no mental health symptoms at intervention T0, a subgroup analysis was conducted on participants who reported at least mild symptoms (score ≥1 on a 0–4 scale) at intervention T0. The results from these symptomatic participants in all groups were pooled together. Domains such as anxiety, worry, irritability, anhedonia and life difficulty feeling, show significant reduction over time of symptoms (Friedman, *p* < 0.05). Significant decrease in anxiety, irritability and life difficulty was found between intervention T0 and interventions T2 (FDR-corrected Wilcoxon: anxiety, *p* = 0.0050, irritability; *p* = 0.0169, life difficulty, *p* = 0.0136). Anxiety, worrying, irritability, anhedonia, and life difficulty scores show a significant decrease at intervention T3, when compared to intervention 0 (FDR-corrected Wilcoxon, *p* = 0.0013, *p* = 0.0055, *p* = 0.0142, *p* = 0.0481, *p* = 0.0308, respectively) (Table 6).

**Table 6:** Impact of scOat fiber on a subgroup of participants with mental health scores ≥1.

Mental health domain	*n*	T0	T2	T3	Friedman’s *p*	Wilcoxon’s *p* (T0 to T2)	Wilcoxon’s *p* (T0 vs T3)
Anxiety (GAD1)	28	1.29 (0.52)	0.71 (0.65)	0.64 (0.85)	**0.0001**	**0.0050**	**0.0013**
Worry (GAD2)	20	1.33 (0.56)	1.10 (0.87)	0.67 (0.84)	**0.0019**	0.4875	**0.0055**
Irritability (GAD6)	29	1.27 (0.57)	0.83 (0.78)	0.83 (0.86)	**0.0005**	**0.0169**	**0.0142**
Anhedonia (PHQ1)	18	1.35 (0.57)	1.05 (0.92)	0.85 (1.01)	**0.0435**	0.1790	**0.0481**
Mood (PHQ2)	15	1.13 (0.33)	0.88 (0.70)	0.50 (0.61)	0.0577	0.6239	0.0624
Concentration (PHQ7)	22	1.26 (0.61)	0.91 (0.83)	0.70 (0.86)	0.0577	0.6239	0.0624
Life difficulty (PHQ10)	20	1.19 (0.39)	0.67 (0.56)	0.71 (0.82)	**0.0009**	**0.0136**	**0.0308**

Values represent mean (SD). Data corresponds to all doses pooled. Bold values indicate significant differences (*p* < 0.05).

In addition, participants completed a weekly questionnaire assessing appetite. Appetite scores remained stable over the 2-week intervention period across all groups (data not shown).

Daily questionnaire data were used to evaluate dietary deviations, change in perceived energy levels (including “morning slumps”), appetite and sleep duration. Daily sleep and appetite metrics did not vary significantly over time or between groups with fiber consumption for 2 weeks (Mann-Kendall, *p* > 0.05). Rates of reporting morning slumps were infrequent, occurring in fewer than 20% of participants in any group.

## 4 Discussion

This study evaluated the tolerability of scOat Fiber in healthy adults. This novel fiber is the product of controlled depolymerization of oat fiber, which significantly increases palatability and solubility of the original fiber. Oat fibers are well known to support healthy blood glucose levels and to have beneficial effects on postprandial blood glucose. However, incorporating enough for meaningful dietary impact requires consumption of large amounts of oat-based food. Approximately 20 g of scOat Fiber can deliver a fiber content equivalent to that found in four servings of oatmeal, offering an alternative approach to achieve similar diet intake and obtain metabolic and digestive benefits of oat fiber. From an organoleptic perspective, scOat Fiber has a broader range of food applications than oat fiber polysaccharide, making it a candidate for broad fiber supplementation. However, the GI tolerance of scOat Fiber was unknown and therefore was evaluated. Three different amounts of fiber were tested for tolerability and safety. The results indicate that scOat Fiber was well tolerated and safe – even at the highest dose tested, 20 g/day. Across all the tested doses, participants reported no increase in GI discomfort, with GSRS total scores remaining in the low range. Reported adverse effects assessed as possibly related to the scOat Fiber were mild, transient, and with no difference between doses.

GI symptoms, in fact, improved in the 5 g/day and 10 g/day groups despite the low level of symptoms at baseline, particularly in relation to overall symptoms (Total GSRS score) and the abdominal pain domain (GSRS abdominal pain). The improvement was seen after 1 week of intervention for the 5 g/day group and after 2 weeks for the 10 g/day group. Improvements were also seen in relation to the constipation domain after 2 weeks. Despite the improvement in constipation symptoms, no increase in diarrhea symptoms was provoked.

These results suggest that scOat Fiber may help address the chronic under-consumption of dietary fiber in the population by providing an alternative form of fiber that is well tolerated even at high doses. Unlike many other fibers, scOat Fiber did not provoke GI intolerance and it was associated with improvement of some GI symptoms. This study counters the common consumer perception that all fibers cause digestive discomfort and highlights that fibers with specific structural characteristics can elicit diverse physiological responses. For example, inulin, which is commonly added to food and beverages, often provokes GI intolerance at doses as low as 7.5 g/day, with studies reporting flatulence and bloating, and abdominal pain (27, 28). Studies with short chain fructans (FOS) also reported intolerance symptoms at doses above 5 g/day (29). Polydextrose has shown significant increase of bloating at doses of 6.25 g/day (30), and resistant dextrin can increase GI symptoms at 7.5 g/day (31). The favorable tolerability of scOat Fiber may be related to its specific structural characteristics and abundance of shorter chain oligosaccharides. β-glucan, the primary component in oat fiber, is predominantly fermented by a narrower subset of gut microbiota compared to other fermentable fibers such as inulin or FOS. This selective fermentation profile may result in a more targeted SCFA production while limiting excessive microbial gas production. *In vivo* fermentation studies have shown that oat β-glucan generates substantially lower gas volumes compared to highly fermentable fibers such as inulin, while still producing acetate, propionate and butyrate (32).

Oat polysaccharides are associated with glycemic and cardiovascular health benefits (16, 33) and therefore the impact of scOat Fiber on postprandial glucose regulation and glycemic control was explored using continuous glucose monitoring. The results suggest that scOat Fiber may have a positive effect on glycemic parameters, particularly at higher daily amounts. Peak glucose after a rice challenge dropped significantly after 2 weeks of scOat Fiber consumption at amounts of 10 g/day and 20 g/day. A similar reduction was obtained for glucose spike height at 20 g/day. Furthermore, consumption of 20 g/day led to an average 13% decrease in peak glucose over 2 weeks, a magnitude of effect that aligns with reduction comparable to the magnitude of postprandial glycemic improvements considered physiologically beneficial in FDA evaluations of non-digestible carbohydrate intervention (34). Contrary to peak glucose, no impact of scOat Fiber on the total exposure to glucose over the 4 h after a rice challenge, as measured by AUC, was seen. A possible explanation is that scOat Fiber may lower the rate of glucose absorption, lowering the glucose peak but leaving the total amount of glucose taken up, unchanged. These findings suggest that the improvement in postprandial glucose metrics reflect more than just modulation of glucose absorption, previously described for scOat fiber (21). A potential mechanism is that scOat Fiber may improve metabolic functioning over time, for example improving insulin sensitivity, allowing for a quicker initial response that lowers peaks but simultaneously reducing glucose troughs.

Peak glucose and AUC represent distinct but complementary aspects of postprandial glucose handling, providing an insight of how scOat Fiber modulates the acute glycemic response to meals. In contrast, parameters like TIR, SD, CV and MAGE reflect broader metabolic regulation, capturing effects beyond a single meal. A potential improvement in metabolic functioning is supported by the results of this study, obtained in relation to these parameters, which are increasingly recognized as risk factors for metabolic syndrome and cardiovascular events (35, 36). The results suggest that scOat Fiber not only minimizes postprandial glucose excursions, reducing the amplitude of glucose spikes, but also may improve basal glycemia, a clinically meaningful effect for metabolic health.

While most studies emphasize β-glucan viscosity as a key mechanism in glycemic control, this study demonstrates, for the first time, that cereal β-glucans improve blood glucose outcomes independent of their viscosity. This study aligns with recent research which indicates that short chain or non-viscous fibers may offer glycemic benefits, especially when controlling for food dose. It has been shown that low molecular weight β-glucan performs similarly to high molecular weight β-glucan on metrics such as iAUC or peak glucose (37). Importantly, a study on evening intake of cereal β-glucan found improvements in the next day’s glycemia despite no clear dependence on viscosity, pointing to a possible gut microbiome-mediated effect (38). Production of SCFA by fiber fermentation may impact glucose dynamics as described before (3), potentially improving glucose handling and insulin sensitivity.

In an exploratory analysis, participants with mild mental symptoms (average score in the baseline mood questionnaire of 1 or higher) reported significant improvement after 2 weeks of consuming scOat Fiber. Consistency of the changes across multiple symptom domains and the statistical significance of the comparisons suggest that scOat Fiber may support mental health and this potential positive effect is worth further study. While these findings were statistically significant and consistent, they must be interpreted as exploratory due to the absence of a control group, pooling of the participants and the small subgroup sample size. Nonetheless, they align with growing evidence in the literature linking fiber intake and mental health, thus future evaluations should be made with participants that present elevated symptom scores following expert recommendations (39). Mechanistically, the link between fiber and mental health is supported by an extensive body of research. SCFAs produced in the gut as a product of fiber fermentation are absorbed and can cross the blood brain barrier, reduce brain inflammation and affect neurotransmitters that regulate mood, such as serotonin, GABA and dopamine (40). In addition, fibers can improve gut barrier function and lower levels of circulating toxins, which may reduce overall inflammation and the impact on gut-brain function (41). Similar benefits have been seen with other fibers, such as psyllium, which has been linked to better mood, and inulin, which may improve both mood and cognitive function (42, 43). There is also evidence that galacto-oligosaccharides (GOS) and resistant starch may offer improvements in overall mental health (44, 45).

Several limitations of the current study should be acknowledged. The open label design and lack of a placebo control limit the ability to distinguish intervention effects from placebo effects, particularly for subjective outcomes such as mood and sleep. Dietary intake was not strictly controlled; however, participants were instructed to maintain their habitual diet, and daily questionaries captured deviation such as unusual meals, which were taken into account during data interpretation. This design was selected to make the results as applicable as possible to the general population but represents a limitation in that dietary confounding aspects cannot be fully excluded. Further, potential confounding factors such as baseline fiber intake, exercise and sleep were not taken into account, and the intervention period was relatively short (14 days), and long-term benefits remain unknown. While multiple glucose challenges were conducted, variability in participant adherence (i.e., exact rice intake timing, fasting) may have introduced noise into the CGM data. In addition, using repeated reference food testing is recommended in glycemic index protocols to reduce intra individual variability. However, because the glycemic challenge was designed as an exploratory assessment of change over time and not to determine an index, a single standardized rice challenge was used at each tested time point. The use of rice challenges over time allows each participant to serve as their own control, which mitigates variability. Time to peak glucose was not evaluated in this study. While this parameter may provide complementary insight into the temporal dynamics of the postprandial glycemic response, it is known to exhibit substantial intra- and inter-individual variability. In the context of the present study design and sample size, magnitude-based metrics were prioritized, as they provide more robust and reproducible measures of glycemic response. Future studies incorporating larger sample sizes and repeated standardized challenge testing may enable a more reliable assessment of time-dependent glycemic parameters.

Stool samples were collected for microbiome and SCFA analysis, these results will be reported at a later time. Future publications integrating these data will help clarify the biological pathways through which scOat Fiber exerts its effects.

Although the current study did not include a placebo control, comparison between dose groups can partially compensate for the absence of a placebo control. Also, the strength and trends of the observed effects on GI symptoms, glucose control and mental symptoms suggest that those effects may be different than a nonspecific placebo response. Future randomized controlled trials are needed to confirm these findings and to better understand dose response effects and underlying mechanisms. The glycemic benefits observed with scOat Fiber are notable in the context of its low viscosity in solution. It is believed that conventional high molecular weight oat β-glucan achieves glycemic benefits largely through its high viscosity, which slows gastric emptying and delays glucose absorption in the small intestine. In contrast, depolymerized short-chain oat β-glucan exhibits minimal viscosity increase and yet demonstrates glycemic benefits, suggesting that viscosity-independent mechanisms, such as inhibition of digestive enzymes, modulation of glucose transporters, and microbiome-mediated SCFA production may play an important role. These properties distinguish scOat Fiber from both viscous oat β-glucan and other soluble fibers such as psyllium, or pectin, which rely on viscosity or alternative fermentation pathways for their glycemic effects. Further comparisons between depolymerized and native oat β-glucan, as well as with other soluble fibers, would be valuable to better delineate these structure–function relationships and relative efficacy. scOat Fiber is stable in food and beverage applications, and easy to incorporate into beverages, food and supplements, without compromising texture or palatability. Being able to incorporate this novel fiber as a food ingredient or supplement is a safe and scalable strategy to incorporate products that can support glycemic and mental well-being.

## 5 Conclusion

As many low tolerability fibers are added to foods or used as supplements, determination of tolerance to novel fibers is important. The findings from this study provide evidence that scOat Fiber, a short chain β-glucan, is safe and well tolerated, even at high daily intakes. This high tolerability allows food formulation with fiber dose levels that can beneficially impact consumers and may help addressing the current “fiber gap” in western diets. scOat Fiber shows functional properties relevant to GI, metabolic and mental health. The results justify further investigation in populations with affected glucose regulation or participants with mild mental health symptoms. Given the global underconsumption of fiber and its implication in health, the development of tolerable and functional fibers represents a significant opportunity to improve public health.

## Data Availability

The raw data supporting the conclusions of this article will be made available by the authors, without undue reservation.

## References

[1] Eswaran S Muir J Chey WD. Fiber and functional gastrointestinal disorders. Am J Gastroenterol. (2013) 108:718–27. doi: 10.1038/ajg.2013.63, 23545709

[2] Scholz-Ahrens KE Ade P Marten B Weber P Timm W Açil Y . Prebiotics, probiotics, and synbiotics affect mineral absorption, bone mineral content, and bone structure. J Nutr. (2007) 137:838S–46S. doi: 10.1093/jn/137.3.838S, 17311984

[3] Slavin J. Fiber and prebiotics: mechanisms and health benefits. Nutrients. (2013) 5:1417–35. doi: 10.3390/nu5041417, 23609775 PMC3705355

[4] Timm DA Thomas W Boileau TW Williamson-Hughes PS Slavin JL. Polydextrose and soluble corn fiber increase five-day fecal wet weight in healthy men and women. J Nutr. (2013) 143:473–8. doi: 10.3945/jn.112.170118, 23427334

[5] McRae MP. Dietary fiber is beneficial for the prevention of cardiovascular disease: an umbrella review of meta-analyses. J Chiropr Med. (2017) 16:289–99. doi: 10.1016/j.jcm.2017.05.005, 29276461 PMC5731843

[6] McRae MP. Dietary fiber intake and type 2 diabetes mellitus: an umbrella review of meta-analyses. J Chiropr Med. (2018) 17:44–53. doi: 10.1016/j.jcm.2017.11.002, 29628808 PMC5883628

[7] Storz MA Ronco AL. Nutrient intake in low-carbohydrate diets in comparison to the 2020–2025 dietary guidelines for Americans: a cross-sectional study. Br J Nutr. (2022) 129:1–14. doi: 10.1017/S0007114522001908, 35730148 PMC9991840

[8] Reynolds A Mann J Cummings J Winter N Mete E Morenga LT. Carbohydrate quality and human health: a series of systematic reviews and meta-analyses. Lancet. (2019) 393:434–45. doi: 10.1016/S0140-6736(18)31809-9, 30638909

[9] Dhingra D Michael M Rajput H Patil RT. Dietary fibre in foods: a review. J Food Sci Technol. (2011) 49:255–66. doi: 10.1007/s13197-011-0365-5 23729846 PMC3614039

[10] Holscher HD. Dietary fiber and prebiotics and the gastrointestinal microbiota. Gut Microbes. (2017) 8:172–84. doi: 10.1080/19490976.2017.1290756, 28165863 PMC5390821

[11] Martins GN Ureta MM Tymczyszyn EE Castilho PC Gomez-Zavaglia A. Technological aspects of the production of fructo and galacto-oligosaccharides. Enzymatic synthesis and hydrolysis. Front Nutr. (2019) 6:78. doi: 10.3389/fnut.2019.00078, 31214595 PMC6554340

[12] Bruhwyler J Carreer F Demanet E Jacobs H. Digestive tolerance of inulin-type fructans: a double-blind, placebo-controlled, cross-over, dose-ranging, randomized study in healthy volunteers. Int J Food Sci Nutr. (2009) 60:165–75. doi: 10.1080/09637480701625697, 18608562

[13] Holscher HD Chumpitazi BP Dahl WJ Fahey GC Liska DJ Slavin JL . Perspective: assessing tolerance to nondigestible carbohydrate consumption. Adv Nutr. (2022) 13:2084–97. doi: 10.1093/advances/nmac091, 36041178 PMC9776727

[14] Arifuzzaman M Won TH Yano H Uddin J Emanuel ER Hu E . Dietary fiber is a critical determinant of pathologic ILC2 responses and intestinal inflammation. J Exp Med. (2024) 221:e20232148. doi: 10.1084/jem.20232148, 38506708 PMC10955042

[15] Arifuzzaman M Won TH Li T-T Yano H Digumarthi S Heras AF . Inulin fibre promotes microbiota-derived bile acids and type 2 inflammation. Nature. (2022) 611:578–84. doi: 10.1038/s41586-022-05380-y, 36323778 PMC10576985

[16] Lazaridou A Biliaderis CG. Molecular aspects of cereal β-glucan functionality: physical properties, technological applications and physiological effects. J Cereal Sci. (2007) 46:101–18. doi: 10.1016/j.jcs.2007.05.003

[17] Tamura K Hemsworth GR Dejean G Rogers TE Pudlo NA Urs K . Molecular mechanism by which prominent human-gut bacteroidetes utilize mixed-linkage beta-glucans, major health-promoting cereal polysaccharides. Cell Rep. (2017) 21:417–30. doi: 10.1016/j.celrep.2017.09.049, 29020628 PMC5656003

[18] European Food Safety Authority. Scientific opinion on the substantiation of health claims related to beta-glucans from oats and barley and maintenance of normal blood LDL-cholesterol concentrations pursuant to article 13(1) of regulation (EC) no 1924/2006. EFSA J. (2011) 9:2207. doi: 10.2903/j.efsa.2011.2207 PMC13129977 42078707

[19] Ren Y Xie H Liu L Jia D Yao K Chi Y. Processing and prebiotics characteristics of β-glucan extract from Highland barley. Appl Sci. (2018) 8:1481. doi: 10.3390/app8091481

[20] Maldonado-Gomez MX Ng KM Drexler RA Conner AMS Vierra CG Krishnakumar N . A diverse set of solubilized natural fibers drives structure-dependent metabolism and modulation of the human gut microbiota. MBio. (2025) 16:e0047025. doi: 10.1128/mbio.00470-25, 40214223 PMC12077125

[21] Marcobal AM McConnell BR Drexler RA Ng KM Maldonado-Gomez MX Conner AMS . Highly soluble β-glucan fiber modulates mechanisms of blood glucose regulation and intestinal permeability. Nutrients. (2024) 16:2240. doi: 10.3390/nu16142240, 39064683 PMC11279855

[22] Svedlund J Sjodin I Dotevall G. GSRS-a clinical rating scale for gastrointestinal symptoms in patients with irritable bowel syndrome and peptic ulcer disease. Dig Dis Sci. (1988) 33:129–34. doi: 10.1007/BF01535722, 3123181

[23] Spitzer RL Kroenke K Williams JB Lowe B. A brief measure for assessing generalized anxiety disorder: the GAD-7. Arch Intern Med. (2006) 166:1092–7. doi: 10.1001/archinte.166.10.1092, 16717171

[24] Kroenke K Spitzer RL Williams JB. The PHQ-9: validity of a brief depression severity measure. J Gen Intern Med. (2021) 16:606–13. doi: 10.1046/j.1525-1497.2001.016009606.x, 11556941 PMC1495268

[25] Garg S Zisser H Schwartz S Bailey T Kaplan R Ellis S . Improvement in glycemic excursions with a transcutaneous, real-time continuous glucose sensor: a randomized controlled trial. Diabetes Care. (2006) 29:44–50. doi: 10.2337/diacare.29.01.06.dc05-1686, 16373894

[26] Akasaka T Sueta D Tabata N Takashio S Yamamoto E Izumiya Y . Effects of the mean amplitude of glycemic excursions and vascular endothelial dysfunction on cardiovascular events in nondiabetic patiens with coronary artery diseases. J Am Heart Assoc. (2017) 26:e004841. doi: 10.1161/JAHA.116.004841, 28446494 PMC5524064

[27] Holscher HD Doligale JL Bauer LL Gourineni V Pelkman CL Fahey GC . Gastrointestinal tolerance and utilization of agave inulin by healthy adults. Food Funct. (2014) 5:1142–9. doi: 10.1039/C3FO60666J, 24664349

[28] Ripoll C Flourié B Megnien S Hermand O Janssens M. Gastrointestinal tolerance to an inulin-rich soluble roasted chicory extract after consumption in healthy subjects. Nutrition. (2010) 26:799–803. doi: 10.1016/j.nut.2009.07.013, 19931416

[29] Bonnema AL Kolberg LW Thomas W Slavin JL. Gastrointestinal tolerance of chicory inulin products. J Am Diet Assoc. (2010) 110:865–8. doi: 10.1016/j.jada.2010.03.025, 20497775

[30] Hull S Re R Tiihonen K Viscione L Wickham M. Consuming polydextrose in a mid-morning snack increases acute satiety measurements and reduces subsequent energy intake at lunch in healthy human subjects. Appetite. (2012) 59:706–12. doi: 10.1016/j.appet.2012.08.004, 22885981

[31] Fastinger ND Karr-Lilienthal LK Spears JK Swanson KS Zinn KE Nava GM . A novel resistant maltodextrin alters gastrointestinal tolerance factors, fecal characteristics, and fecal microbiota in healthy adult humans. J Am Coll Nutr. (2008) 27:356–66. doi: 10.1080/07315724.2008.10719712, 18689571

[32] Carlson JL Erickson JM Hess JM Gould TJ Slavin JL. Prebiotic dietary fiber and gut health: comparing the in vitro fermentations of beta-glucan, inulin and xylooligosaccharide. Nutrients. (2017) 9:1361. doi: 10.3390/nu9121361, 29244718 PMC5748811

[33] Tosh SM. Review of human studies investigating the post-prandial blood-glucose lowering ability of oat and barley food products. Eur J Clin Nutr. (2013) 67:310–7. doi: 10.1038/ejcn.2013.25, 23422921

[34] U.S. Food and Drug Administration. In: U.S. Food and Drug Administration, editor. Review of the Scientific Evidence on the Physiological Effects of Certain Non-Digestible Carbohydrates. Silver Spring, MD: (2024)

[35] Belli M Bellia A Sergi D Barone L Lauro D Barillà F. Glucose variability: a new risk factor for cardiovascular disease. Acta Diabetol. (2023) 60:1291–9. doi: 10.1007/s00592-023-02097-w, 37341768 PMC10442283

[36] Guo W Song Y Sun Y Du H Cai Y You Q . Systemic immune-inflammation index is associated with diabetic kidney disease in type 2 diabetes mellitus patients: evidence from NHANES 2011–2018. Front Endocrinol. (2022) 13:1071465. doi: 10.3389/fendo.2022.1071465, 36561561 PMC9763451

[37] Ames N Malunga LN Mollard R Johnson J Chu Y Thandapilly SJ. Effect of processing on oat β-glucan viscosity, postprandial glycemic response and subjective measures of appetite. Food Funct. (2021) 12:3672–9. doi: 10.1039/D0FO03283B, 33900322

[38] Telle-Hansen VH Gaundal L Høgvard B Ulven SM Holven KB Byfuglien MG . A three-day intervention with granola containing cereal beta-glucan improves glycemic response and changes the gut microbiota in healthy individuals: a crossover study. Front Nutr. (2022) 9:796362. doi: 10.3389/fnut.2022.796362, 35578615 PMC9106798

[39] Dalile B Boyle NB Ruiz FT Chakrabarti A Respondek F Dodd GF . Targeting cognitive resilience through prebiotics: a focused perspective. Adv Nutr. (2025) 16:100343. doi: 10.1016/j.advnut.2024.100343, 39551433 PMC11663957

[40] Wolever TMS Rahn M Dioum EH Jenkins AL Ezatagha A Campbell JE . Effect of oat β-glucan on affective and physical feeling states in healthy adults: evidence for reduced headache, fatigue, anxiety and limb/joint pains. Nutrients. (2021) 13:1534. doi: 10.3390/nu13051534, 34062937 PMC8147290

[41] Kelly JR Kennedy PJ Cryan JF Dinan TG Clarke G Hyland NP. Breaking down the barriers: the gut microbiome, intestinal permeability and stress-related psychiatric disorders. Front Cell Neurosci. (2015) 9:392. doi: 10.3389/fncel.2015.00392, 26528128 PMC4604320

[42] Schmidt K Cowen PJ Harmer CJ Tzortzis G Errington S Burnet PWJ. Prebiotic intake reduces the waking cortisol response and alters emotional bias in healthy volunteers. Psychopharmacology. (2015) 232:1793–801. doi: 10.1007/s00213-014-3810-0, 25449699 PMC4410136

[43] Johnstone N Cohen Kadosh K. Indicators of improved emotion behavior in 6–14-year-old children following a 4-week placebo controlled prebiotic supplement intervention at home with a parent. Nutr J. (2025) 24:34. doi: 10.1186/s12937-025-01098-5, 40025494 PMC11871729

[44] Kadyan S Park G Hochuli N Miller K Wang B Nagpal R. Resistant starches from dietary pulses improve neurocognitive health via gut-microbiome-brain axis in aged mice. Front Nutr. (2024) 11:1322201. doi: 10.3389/fnut.2024.1322201, 38352704 PMC10864001

[45] Johnstone N Milesi C Burn O van den Bogert B Nauta A Hart K . Anxiolytic effects of a galacto-oligosaccharides prebiotic in healthy females (18–25 years) with corresponding changes in gut bacterial composition. Sci Rep. (2021) 11:8302. doi: 10.1038/s41598-021-87865-w, 33859330 PMC8050281

