# Supplementary material for: Short-chain oat fiber improves gastrointestinal tolerance and regulates glucose metabolism: a two-week open-label study in healthy adults
Source: Front Nutr. 2026 Jun 10;13:1745303. doi: 10.3389/fnut.2026.1745303 (PMC13290552; doi:10.3389/fnut.2026.1745303)
Supplement: Supplementary file 2 [file Table_2.DOCX]

Supplementary Material

**Supplementary Table S2.** Adverse events

| **AE Type** | **Total Count of AE Occurrences** | **Count of participants impacted** | **Impacted in 5 g/day group** | **Impacted in 10 g/day group** | **Impacted in 20 g/day group** |
| --- | --- | --- | --- | --- | --- |
| Gas | 30 | 26 | 11 | 9 | 6 |
| Bloating | 17 | 15 | 7 | 4 | 4 |
| Constipation | 15 | 13 | 4 | 4 | 5 |
| Abdominal pain | 7 | 7 | 4 | 1 | 2 |
| Diarrhea | 5 | 5 | 4 | 0 | 1 |
| Dizziness | 2 | 2 | 1 | 1 | 0 |
| Borborygmus | 1 | 1 | 0 | 0 | 1 |
| Fatigue | 1 | 1 | 1 | 0 | 0 |
| Loose Stool | 1 | 1 | 0 | 1 | 0 |
| Nausea | 1 | 1 | 1 | 0 | 0 |
| Reflux | 1 | 1 | 0 | 1 | 0 |
